# SARS-CoV-2 Spike protein activates TMEM16F-mediated platelet procoagulant activity

**DOI:** 10.3389/fcvm.2022.1013262

**Published:** 2023-01-04

**Authors:** Ambra Cappelletto, Harriet E. Allan, Marilena Crescente, Edoardo Schneider, Rossana Bussani, Hashim Ali, Ilaria Secco, Simone Vodret, Roberto Simeone, Luca Mascaretti, Serena Zacchigna, Timothy D. Warner, Mauro Giacca

**Affiliations:** ^1^British Heart Foundation Centre of Research Excellence, School of Cardiovascular Medicine & Sciences, King’s College London, London, United Kingdom; ^2^Barts and the London School of Medicine and Dentistry, Blizard Institute, Queen Mary University of London, London, United Kingdom; ^3^Department of Medical, Surgical and Health Sciences, University of Trieste, Trieste, Italy; ^4^International Centre for Genetic Engineering and Biotechnology (ICGEB), Trieste, Italy; ^5^Dipartimento di Medicina Trasfusionale Giuliano-Isontino, Azienda Sanitaria Universitaria Giuliano Isontina (ASUGI), Trieste, Italy

**Keywords:** platelets, SARS-CoV-2, TMEM16F, coagulation, Niclosamide, Clofazimine, COVID-19, Spike

## Abstract

Thrombosis of the lung microvasculature is a characteristic of COVID-19 disease, which is observed in large excess compared to other forms of acute respiratory distress syndrome and thus suggests a trigger for thrombosis that is endogenous to the lung. Our recent work has shown that the SARS-CoV-2 Spike protein activates the cellular TMEM16F chloride channel and scramblase. Through a screening on >3,000 FDA/EMA approved drugs, we identified Niclosamide and Clofazimine as the most effective molecules at inhibiting Spike-induced TMEM16 activation. As TMEM16F plays an important role in stimulating the procoagulant activity of platelets, we investigated whether Spike directly affects platelet activation and pro-thrombotic function and tested the effect of Niclosamide and Clofazimine on these processes. Here we show that Spike, present either on the virion envelope or on the cell plasma membrane, promotes platelet activation, adhesion and spreading. Spike was active as a sole agonist or, even more effectively, by enhancing the function of known platelet activators. In particular, Spike-induced a marked procoagulant phenotype in platelets, by enhancing Ca^2+^ flux, phosphatidylserine externalization on the platelet outer cell membrane, and thrombin generation. Eventually, this increased thrombin-induced clot formation and retraction. Both Niclosamide and Clofazimine blocked this Spike-induced procoagulant response. These findings provide a pathogenic mechanism to explain lung thrombosis-associated with severe COVID-19 infection. We propose that Spike, present in SARS-CoV-2 virions or exposed on the surface of infected cells in the lungs, enhances the effects of inflammation and leads to local platelet stimulation and subsequent activation of the coagulation cascade. As platelet TMEM16F is central in this process, these findings reinforce the rationale of repurposing Niclosamide for COVID-19 therapy.

## 1 Introduction

Thrombosis is a defining characteristic of advanced COVID-19 lung disease. Recent meta-analyses reveal that elevated D-dimers, fibrinogen and thrombosis-associated biomarkers are present in over 20% of patients with COVID-19 and in at least 50% of patients requiring intensive care (1–5). At the histological level, our own post-mortem analysis of 41 patients with COVID-19 revealed the presence of microthrombosis in the lungs of 83% of patients requiring intensive care and 75% of all the other patients (6). This high prevalence resonates with that of several other recent pathology investigations (7–11). While a thrombotic response is common in other causes of acute respiratory distress syndrome (ARDS), the magnitude of this response appears a hallmark of COVID-19. A post-mortem study reported that severe vascular injury, including alveolar microthrombi, was nine times more prevalent in COVID-19 lungs than in patients with influenza (7).

At least three observations suggest that lung thrombosis in COVID-19 is triggered by local events. First, thrombi and fibrin deposition in the lungs are asynchronous, by which recent thrombi infiltrated by inflammatory cells are close to older thrombi, in advanced stage of fibrotic organization (6). Second, the presence of macro- or micro-vascular thrombosis is only sporadically detected in other organs (6–11). Third, patients with severe COVID-19 have high levels of D-dimer and fibrinogen, but do not show an increase in prothrombin time or a decrease in antithrombin levels and rarely develop disseminated intravascular coagulation (12); this argues against thrombosis as a consequence of a systemic consumptive coagulopathy.

This pulmonary intravascular coagulopathy (13) has variously been attributed to endothelial dysfunction (6, 14), or the hyperinflammatory state that accompanies SARS-CoV-2 infection (15–19), or immunothrombosis, by which neutrophils and monocytes activate the coagulation cascade as a host immune defense against infection (20–22), including the release of neutrophil extracellular traps (NETs) (23, 24). While these mechanisms most likely contribute to the local pro-thrombotic state, they still do not explain why lung thrombosis is particularly frequent in COVID-19 compared to other causes of ARDS.

Several observations point to a specific involvement of platelets in the pathogenesis of COVID-19 thrombosis. A common characteristic of patients with advanced COVID-19 disease is thrombocytopenia (5, 25–27); COVID-19 lungs show increased number of pulmonary megakaryocytes, which could be indicative of increased local megakaryopoiesis in response to platelet consumption (28); SARS-CoV-2 infection is associated with platelet hyperreactivity, including increased P-selectin expression, platelet activation and spreading on both fibrinogen and collagen, and elevated levels of circulating platelet-neutrophil, -monocyte, and -T-cell aggregates (29–32). The actual molecular mechanisms leading to this platelet hyperactivity during SARS-CoV-2 infection, coupled with local thrombosis, remain largely elusive and is likely multifactorial.

In the course of SARS-CoV-2 infection, the viral envelope Spike protein exerts a fundamental function by mediating virus entry into the cells. In particular, the Spike surface unit (S1) binds the ACE2 cellular receptor (33), which facilitates docking of the virion onto the surface of the target cells. A subsequent activation step carried out by a cellular protease cleaves Spike at the S1/S2 boundary and thus exposes the S2 portion, which promotes membrane fusion and virion content internalization. In the case of SARS-CoV-2, the protease priming event and subsequent fusion can occur at the plasma membrane thanks to the involvement of the TMPRSS2 (33) or furin (34, 35) proteases, hence the capacity of virus-infected cells, which expose Spike on their surface, to form large syncytia with other infected or non-infected cells that express the ACE2 receptor.

While studying the molecular properties of SARS-CoV-2 Spike, we discovered a novel mechanism that regulates Spike-mediated cellular syncytia formation. We screened two libraries of EMA/FDA-approved small molecules to search for drugs that inhibit syncytia formation (36). As the most effective drugs, this screening identified Niclosamide, a synthetic salicylanilide developed in the 1950s as a molluscicide against snails (37) and later approved for tapeworm infection, and Clofazimine, an antibiotic used for the combination treatment of leprosy (38) and, more recently, for drug-resistant tuberculosis (39). Of relevance for platelets, we discovered that Niclosamide blocks the formation of syncytia by inhibiting the cellular Ca^2+^-dependent chloride channel and scramblase TMEM16F, thus preventing the externalization of phosphatidylserine (PS) onto the outer leaflet of the cell plasma membrane (36). TMEM16F is essential for lipid scrambling in platelets during blood coagulation (40, 41), as externalized PS serves as an anchoring site for the assembly of the tenase and prothrombinase complex, which jointly enhance the rate of thrombin generation by several orders of magnitude (42).

Based on these observations, it was tempting for us to speculate that Spike-driven activation of platelets in SARS-CoV-2 infected lungs could be causally involved in the thrombotic process. Here we show that Spike, exposed on either the envelope of virions or the surface of cells, induces a procoagulant phenotype in platelets, which is blocked by both Niclosamide and Clofazimine.

## 2 Materials and methods

### 2.1 Human blood collection and isolation of platelets

All investigations in this study conform to the principles outlined in the Declaration of Helsinki. All human subjects provided their informed written consent.

Studies using human platelets were approved by St Thomas’s Hospital, London, UK Research Ethics Committee (ref. 07/Q0702/24). Blood was collected by venepuncture into tri-sodium citrate (3.2%; Sigma-Aldrich, Burlington, MA, United States) from healthy volunteers (aged 25–40), who had abstained from non-steroidal anti-inflammatory drug consumption for the preceding 14 days. Platelet rich plasma (PRP) was obtained by centrifugation of whole blood (175 × *g*, 15 min, 25°C). Further purification of platelets was achieved by centrifugation of PRP at 1,000 × *g* for 10 min at 25°C in the presence of apyrase (0.02 U/mL, Sigma-Aldrich, Burlington, MA, United States) and prostacyclin (PGI_2_; 2 μM, Tocris Bioscience, Bristol, United Kingdom) followed by resuspension in modified Tyrode’s HEPES buffer (134 mmol/L NaCl, 2.9 mM KCl, 0.34 mmol/L Na_2_HPO_4_, 12 mmol/L NaHCO_3_, 20 mmol/L HEPES, and 1 mmol/L MgCl_2_; pH 7.4; Sigma-Aldrich, Burlington, MA, United States) with glucose (0.1% w/v; Sigma-Aldrich, Burlington, MA, United States) and bovine serum albumin (Sigma-Aldrich, Burlington, MA, United States). Washed platelets were adjusted to a concentration of 3 × 10^8^/ml, allowed to rest of 30 min and then supplemented with 2 mM CaCl_2_ (Sigma-Aldrich, Burlington, MA, United States).

### 2.2 Patients

Patients’ lung samples were obtained from post-mortem analysis of a cohort of patients who died of COVID-19 at the University Hospital in Trieste, Italy, after intensive care support (6). All patients scored positive for SARS-CoV-2 by RT-PCR tests on nasopharyngeal swab and presented symptoms (fever, cough, and dyspnea) as well as imaging data indicative of interstitial pneumonia related to COVID-19 disease. All patients eventually died of clinical acute respiratory distress syndrome related to COVID-19 infection. Further clinical details on these patients are reported in Bussani et al. (6). The use of these samples was approved by the Joint Ethical Committee of the Regione Friuli Venezia Giulia, Italy (re. 0019072/P/GEN/ARCS).

### 2.3 Cell culture

HEK-293T and Vero cells were cultured in Dulbecco’s Modified Eagle Medium (DMEM, Sigma-Aldrich, Burlington, MA, United States, 4.5 g/L) supplemented with 10% fetal bovine serum (FBS, Sigma-Aldrich, Burlington, MA, United States).

### 2.4 Pseudotyped viral particles

For Spike pseudotyped lentiviral particle production, we generated the expression plasmid pEC120-S-D19-V5, in which the 19aa at the Spike C terminus, acting as an ER-retention signal (43), are replaced by a V5 epitope tag. The modified DNA segment was obtained by recombinant PCR and cloned into the pEC117-Spike-V5 vector (36). Pseudotyped viral particles were produced by transfecting HEK293T cell (2.5 × 10^6^) in a 100 mm-dish with 10 μg pLVTHM/GFP (Addgene #12247), 7.5 μg psPAX2 (Addgene #12260), and 6 μg pMD2.G (Addgene #12259) or pEC120-S-D19-V5. The culture medium was collected after 48 h and concentrated three times using Vivaspin columns with 100 kDa cutoff (GE HealthCare, Chicago, IL, United States, #28932363). Aliquots were stored at −80°C.

For titration, RNA from pseudoparticle preparations was isolated using the Viral RNA isolation Kit (Takara, Tokyo, Japan). Viral RNA genome content was quantified using the Lenti-X qRT-PCR Titration Kit (Takara, Tokyo, Japan) and the Quant-X One-Step qRT-PCR TB Green Kit (Takara, Tokyo, Japan).

To assess pseudoparticle infectivity, HEK293T cells were bulk transfected with an ACE2-expressing plasmid and then seeded in a 96-well plate (3 × 10^3^ cells per well). The subsequent day, 5 μL viral particles carrying the G-protein of the Vesicular Stomatitis Virus (VSV-G) or 10 μL particles carrying SARS-CoV-2 Spike were added to each well [the difference in volume was meant to partially compensate for the different transduction efficiency of the two viral preparations (44)]. The plate was fixed at 48 h, stained with Hoechst (Invitrogen, Carlsbad, CA, United States) and imaged.

### 2.5 Platelet aggregation with pseudotyped viral particles and cells

Platelet aggregation was performed as previously described (45) with minor modifications. Briefly, washed platelets were incubated with VSV-G or Spike pseudoparticles (1:10) for 10 min followed by stimulation with collagen related peptide (CRP; 0.3 μg/mL), collagen (0.3 μg/mL, Takeda, Tokyo, Japan) or vehicle. Plates were shaken for 5 min (1,200 rpm, 37°C; BioShake IQ, QInstruments, Jena, Germany), and absorbance was measured at 595 nm using a microplate reader (Sunrise, Tecan, Männedorf, Switzerland). Aggregation was calculated as percentage change in absorbance.

For platelet aggregation on Spike-expressing cells, Vero cells were kept in culture in DMEM supplemented with 10% FBS and transfected with a plasmid coding for either GFP (pZac-GFP) or V5-tagged Spike (pSARS-COV-2-S). After detachment, 20,000 cells were co-incubated with 5 × 10^5^ pre-labeled platelets (1:2,000 Cell Tracker Deep Red Dye, Thermo Fisher Scientific, Waltham, MA, United States) in 96-well plates, and shaken at 200 rpm for 10 min at 37°C. The plate was centrifuged at 300 *g* for 10 min and fixed by 4% final paraformaldehyde (PFA, VWR, Radnor, PA, United States). The plate was permeabilized with 0.5% Triton (Sigma-Aldrich, Burlington, MA, United States) for 10 min, blocked with 1% BSA for 1 h at room temperature (RT) and stained with anti-GFP (1:1,000) or anti-V5 tag (1:500) antibodies for 2 h at RT. After washing three times with 1xPBS, anti-rabbit 488 or anti-mouse 488 secondary antibodies (1:500) were incubated for 1 h at room temperature. Then, plates were washed three times with 1xPBS and incubated with Cell Mask (1:2,000; Thermo Fisher Scientific, Waltham, MA, United States) for 30 min at RT. After further washing with 1xPBS, Hoechst (1:5,000, Thermo Fisher Scientific, Waltham, MA, United States) was added. Images (20 × magnification) were acquired using a Perkin Elmer Operetta CLS High Content Fluorescent microscope. Analysis was performed using the ImageJ software (Fiji).

### 2.6 Platelet adhesion and spreading

Washed platelets (3 × 10^8^/mL) were allowed to adhere to type I Horm collagen (100 μg/mL; Takeda, Tokyo, Japan) coated plates, for 90 min at 37°C. Samples were fixed with 0.2% PFA for 10 min followed by permeabilization with 0.2% Triton (Sigma-Aldrich, Burlington, MA, United States) for 5 min. Samples were washed with 1xPBS and stained with AlexaFluor 488 Phalloidin (1:2,000). Images (63 × magnification) were acquired using a Perkin Elmer Operetta CLS High Content Fluorescent microscope. Analysis was performed using the ImageJ software (Fiji).

### 2.7 Flow cytometry

For externalized phosphatidylserine (PS) analysis, washed platelets were incubated with VSV-G or Spike pseudoparticles (1:10) for 10 min followed by activation with collagen (30 μg/ml) and thrombin (0.5 units, Sigma-Aldrich, Burlington, MA, United States) or vehicle for 15 min (350 rpm, 37°C; BioShake IQ). Platelets were stained with Annexin V-Pacific Blue (1:50; BioLegend, San Diego, CA, United States) and CD61-APC (1:100; BioLegend, San Diego, CA, United States) in annexin binding buffer for 20 min (350 rpm, 37°C; BioShake IQ). For Ca2^+^ levels analysis, washed platelets were stained with Fluo-4 AM (5 μM, Thermo Fisher Scientific, Waltham, MA, United States) for 30 min at 37°C followed by incubation with pseudoparticles (1:10) for 10 min. Platelet samples were activated with vehicle or collagen (30 μg/ml) for 15 min.

In both cases, samples were diluted with modified Tyrode’s HEPES buffer and analyzed on the ACEA Novocyte 3,000 (ACEA Biosciences, San Diego, CA, United States). Analysis was performed using the FlowJo software v.10 (TreeStar, Ashland, OR, United States).

### 2.8 Thrombin measurement

Thrombin activity was measured using a Thrombin Activity Assay Kit (Abcam, Cambridge, United Kingdom). Briefly, PRP was incubated with VSV-G or Spike pseudoparticles (1:10) for 10 min at 37°C, followed by stimulation with collagen (30 μg/ml) for 20 min (350 rpm, 37°C). PRP was diluted 1:10 with thrombin assay buffer, and 50 μl of thrombin substrate was added to each well. Thrombin activity was assessed by measuring the conversion of thrombin substrate into its fluorogenic state using a CLARIOstar fluorescent plate reader (BMG Labtech, Ortenberg, Germany) with 350/450 nm excitation and emission filter. Analysis was performed using MARS analysis software.

### 2.9 Clot retraction

Platelet rich plasma (PRP) was diluted 1:1 with modified Tyrode’s HEPES buffer, supplemented with CaCl_2_ (2 mM) and 10 μl of whole blood. PRP was incubated with VSV-G (1:10), Spike pseudoparticles (1:10), His-tag recombinant SARS-CoV-2 Spike Protein S1/S2 (S-ECD) (1 ng/ml; Thermo Fisher Scientific, Waltham, MA, United States; aa11-1208; RP-87680) or Flag-tag recombinant SARS-CoV-2 Spike RBD (1 ng/ml; Bio-Techne, Minneapolis, MN, United States, 10689-CV-100) for 10 min at 37°C, followed by stimulation with thrombin (0.5 units). Clot retraction was measured over 90 min, taking an image every 15 min. Analysis was performed using ImageJ (NIH).

### 2.10 Effect of drugs on Spike-induced platelet function

The effect of drugs was tested in the presence of 1 μM Niclosamide (Sigma-Aldrich, Burlington, MA, United States) or 5 μM Clofazimine (Sigma-Aldrich, Burlington, MA, United States) for 10 min. Niclosamide (Sigma-Aldrich, Burlington, MA, United States, N0560000) and Clofazimine (Sigma-Aldrich, Burlington, MA, United States, Y0000313) stocks were prepared at 10 mM by resuspending in DMSO. Fresh dilutions were prepared for each experiment. In all the experiments, DMSO (vehicle) was used in controls at the same concentration as in the drug treated samples.

### 2.11 Statistical analysis

Statistical analyses were performed using GraphPad Prism 9.0. Unless stated, data followed a Gaussian distribution. Statistical significance between pairs was determined using unpaired Student’s *t*-test, two tails; statistical significance among three groups of more was determined with one-way ANOVA, two tails, with Dunnett’s *post-hoc* analysis for multiple comparisons.

### 2.12 Antibodies

Immunofluorescence analysis was performed for actin (AlexaFluor 488 Phalloidin, #A12379, Thermo Fisher Scientific, Waltham, MA, United States), GFP (#ab6556, Abcam, Cambridge, United Kingdom) V5 tag (#37-7,500, Thermo Fisher Scientific, Waltham, MA, United States), TMEM16F (ab256302, Abcam, Cambridge, United Kingdom), and ACE2 (ab87436 and ab15348, both from Abcam, Cambridge, United Kingdom). Fluorescent secondary antibodies were obtained from Sigma-Aldrich, Burlington, MA, United States. Immunohistochemistry for platelets was performed with antibody 760–4,249 (Roche, Basel, Switzerland) against CD61.

Immunoblots were performed with primary antibodies against Spike (#GTX632604, Genetex, San Antonio, TX, United States), TMEM16F (#HPA038958, Sigma-Aldrich, Burlington, MA, United States) and tubulin (Cell Signaling, #3873S). Anti-rabbit and anti-mouse HRP-conjugated antibody were obtained from Abcam, Cambridge, United Kingdom.

### 2.13 Immunohistochemistry

Post-mortem samples from patients were fixed in 10% formalin and embedded in paraffin. Antigen retrieval and antibody pre-treatment were performed according to the manufacturer’s instructions. Immunohistochemistry for platelets (CD61 antigen) was performed on one-micron sections. Tissue staining was with Bluing Reagent (Ventana 760–2,037).

### 2.14 Immunoblotting

To detect Spike in pseudoparticles, concentrated Spike and VSV-G pseudoparticles (40 μL) were diluted in 4× protein loading dye. For the detection of TMEM16F by immunoblotting, platelets (9 × 10^8^/mL) were lysed in 2% SDS and quantified by using the Pierce™ BCA Protein Assay Kit (Thermo Fisher Scientific, Waltham, MA, United States). Samples (15–20 μg) were resolved by electrophoresis in 4–20% gradient polyacrylamide gels (Mini-PROTEAN, Bio-Rad, Hercules, CA, United States) and transferred to Trans-blot (Bio-Rad, Hercules, CA, United States). Membranes were blocked in TBST (PBS + 0.1% Tween-20) with 5% skim milk (#9999, Cell Signaling) for 1 h at room temperature. Blots were then incubated (4°C, overnight) with primary antibodies against Spike (1:1,000), TMEM16F (1:1,000) or tubulin (1:10,000). Blots were then washed three times with TBST. Membranes were incubated for 1 h at room temperature with anti-rabbit HRP-conjugated antibody (1:5,000) or anti-mouse HRP-conjugated antibody (1:10,000). After washing three times with TBST (10 min each), blots were developed with ECL (Amersham).

### 2.15 Real-time PCR

RNA was extracted from washed platelets lysed with Trizol and purified using the RNeasy kit from Qiagen, Hilden, Germany (ID: 74004), following the manufacturer’s instruction. cDNA was obtained by retrotranscribing 0.5 μg of RNA. ACE2 expression was evaluated using primer pair: Forward primer CAAGAGCAAACGGTTGAACAC; Reverse primer CCAGAGCCTCTCATTGTAGTCT. The results shown refer to the sequences published in Manne et al. (31).

### 2.16 Data sharing

For original data, please contact Professor MG (mauro.giacca@kcl.ac.uk) or Professor TW (t.d.warner@qmul.ac.uk).

## 3 Results

### 3.1 SARS-CoV2 Spike potentiates platelet aggregation and adhesion

**FIGURE 1:**
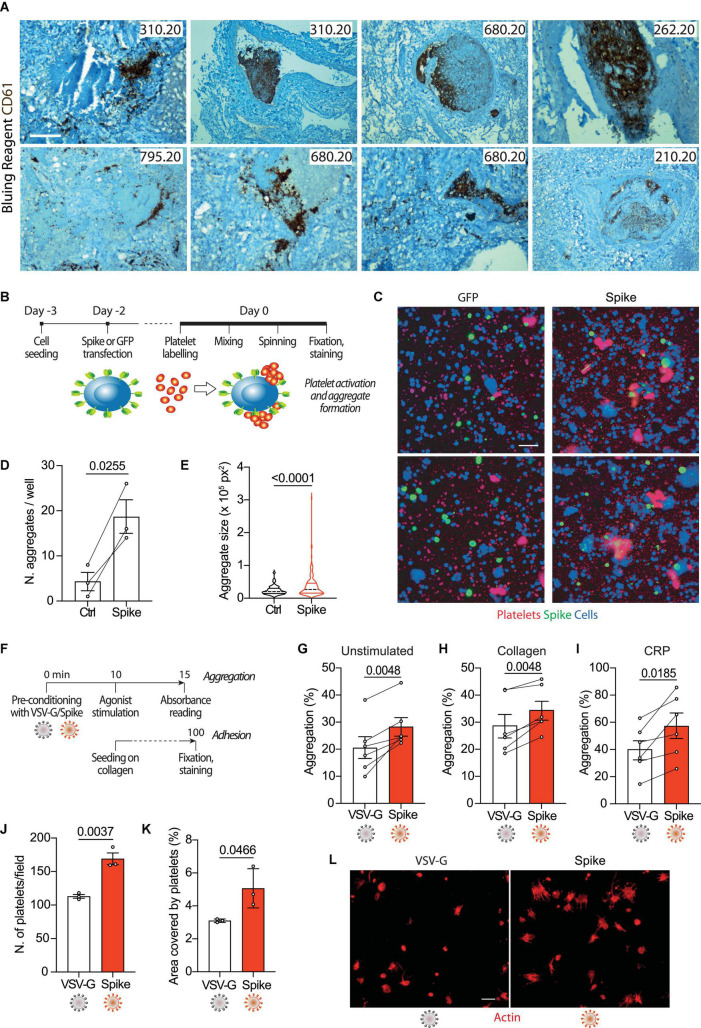
Spike enhances platelet activation. **(A)** Histopathological evidence of platelet aggregates in the thrombotic microvasculature of SARS-COV-2-infected lungs from four COVID-19 patients. Numeric codes identify patients. Platelets were stained using an anti-CD61 glycoprotein antibody. Scale bar: 100 μm. **(B)** Experimental scheme to study platelet activation and aggregate formation. Vero cells transfected to express either Green Fluorescent Protein (GFP) or SARS-CoV-2 Spike were incubated with pre-labeled washed platelets and shaken at 200 rpm for 10 min at 37°C. The plate was centrifuged, fixed, and stained with Cell Mask and antibodies recognizing either GFP or Spike. **(C)** Representative images showing platelet aggregates. Vero cells stained with Cell Mask are in blue; labeled platelets are in red; GFP or Spike are in green. Scale bar, 20 μm. **(D)** Number of aggregates larger than 40,000 px^2^. Results are from *n* = 3 independent experiments. Data are mean ± SEM, statistical significance is indicated (paired Student’s *t*-test). **(E)** Violin plot showing the size of the aggregates found in all the experiments performed. Statistical significance is indicated (unpaired Student’s *t*-test). **(F)** Experimental scheme for platelet activation in suspension. **(G–I)** Percentage of platelet aggregation when incubated with vehicle **(G)**, stimulated with collagen **(H)** or CRP **(I)**. Results are from *N* = 6 independent experiments. Data are mean ± SEM statistical significance is indicated (paired Student’s *t*-test). **(J)** Number of adherent platelets per field. Results are from *n* = 3 independent experiments each performed in duplicate. Each dot represents the mean of six images quantified. Data are mean ± SEM. Statistical significance is indicated (paired Student’s *t*-test). **(K)** Percentage of the area covered by adherent platelets. Results are from *n* = 3 independent experiments performed in duplicate; each dot represents the mean of six images quantified. **(L)** Representative images of platelets adhering on collagen. Images were acquired using a high content fluorescent microscope followed by analysis using the ImageJ software (Fiji). Platelets were stained with F-actin (in red). Scale bar, 5 μm.

COVID-19 lung pathology is characterized by extensive local thrombosis. Our own analysis of post-mortem samples from patients who died of COVID-19 indicated that thrombosis of the lung micro- and macro- vasculature was present in 29/41 (71%) of all patients and in 83% of patients in intensive care units (6). Staining of lung sections with an antibody recognizing the platelet CD61 glycoprotein revealed that these thrombi are massively infiltrated by aggregated platelets (representative images for five patients in Figure 1A).

To understand whether platelet activation could be induced by SARS-CoV-2 Spike on the plasma membrane of infected cells, we incubated Vero cells transfected to express Spike (Wuhan strain) or GFP control with washed human platelets (1 × 10^5^), which had previously been stained with the lipophilic dye CellTracker (scheme in Figure 1B). Transfection efficiency was verified by immunostaining for the transgenes (>40% efficiency in both cases). We observed that the cells expressing Spike significantly increased both the number of platelet aggregates and their overall aggregate area (Figures 1D, E, respectively; *P* < 0.001 in both cases; representative images in Figure 1C).

As far as the receptor that mediates these Spike activities in platelets, we could detect the ACE2 mRNA in platelets of three normal donors that we analyzed, albeit at levels that were lower than those of respiratory Calu-3 cells (Supplementary Figure 1A). When platelets of two of these donors were assessed by immunofluorescence, both ACE2 (visualized with two different antibodies) and TMEM16F were readily detectable (Supplementary Figure 1B).

Next, we wanted to assess whether Spike could also activate platelets in a cell-free context. We generated GFP-expressing lentiviral vectors pseudotyped with either SARS-CoV-2 Spike (Δ19) or the vascular stomatitis virus (VSV) G protein as a control. The vector preparations had comparable genome titres (Supplementary Figure 1C) and Spike was detected in the vector lysates by immunoblotting (Supplementary Figure 1D). Both pseudotyped preparations were effective at transducing HEK293/ACE2 reporter cells (quantification and representative images in Supplementary Figures 1E, F). As indicators for activation, we measured platelet aggregation and adhesion (scheme in Figure 1F). The Spike pseudoparticles significantly enhanced platelet aggregation (from 21 ± 4% in VSV-G controls to 28 ± 3% in Spike-treated platelets; *n* = 6, *P* < 0.01; Figure 1G). There was no effect of VSV-G particle-treated compared to untreated platelets (Supplementary Figures 1G, H). Spike pseudotyped virions also increased aggregation induced by either collagen (29 ± 4 vs. 34 ± 3%; *n* = 6, *P* < 0.01 in VSV-G vs. Spike; *n* = 6, *P* < 0.01) or collagen-related peptide (CRP; 40 ± 7 vs. 57 ± 9%; *n* = 6, *P* < 0.01; Figures 1H, I, respectively).

In addition to potentiating platelet aggregation, Spike also increased platelet adhesion and spreading. The Spike viral particles augmented both the number of platelets adhering on collagen (113 ± 3 vs. 169 ± 9 platelets per field in VSV-G vs. Spike; *n* = 3, *P* < 0.01) and the area covered by the adherent platelets (3% vs. 5 ± 1% in VSV-G vs. Spike; *n* = 3, *P* < 0.05); Figures 1J, K, respectively; representative images in Figure 1L. Also in this case, there was no significant difference between VSV-G particle-treated and untreated platelets (Supplementary Figure 1I).

Taken together, these observations indicate that Spike on either the virion envelope or the cell plasma membrane enhances platelet aggregation and adhesion.

### 3.2 SARS-CoV2 Spike induces a procoagulant response in platelets

**FIGURE 2:**
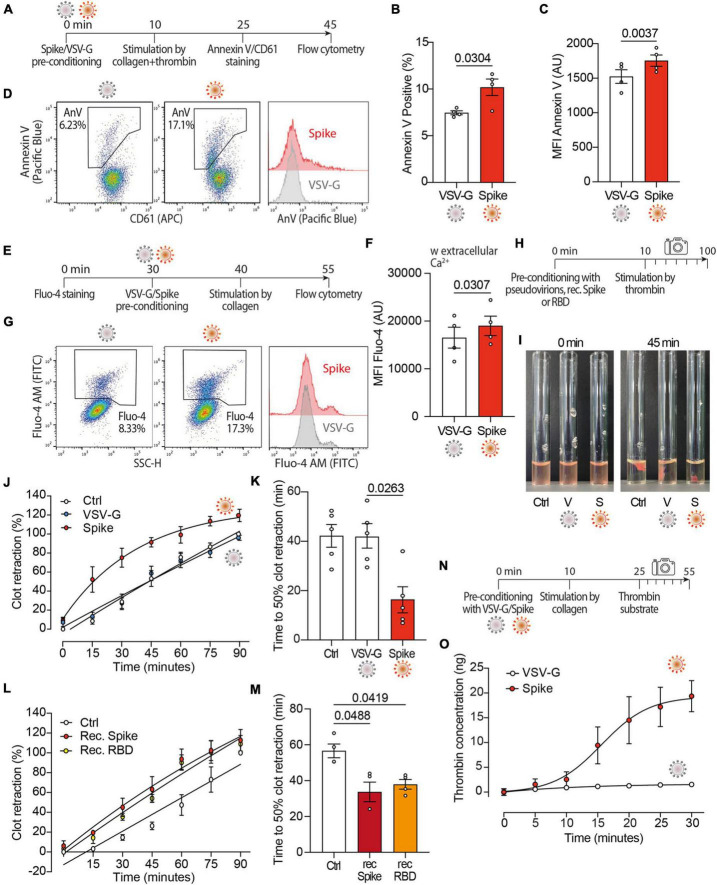
Spikes activates the procoagulant activity of platelets. **(A)** Experimental scheme to study platelet activation by pseudovirions. Washed platelets were incubated with 1:10 diluted VSV-G or Spike for 10 min, followed by incubation with collagen (30 μg/ml) and thrombin (0.5 units) for 15 min. Platelets were stained with Annexin V-Pacific Blue and CD61-APC for 20 min at 37°C and then analyzed by flow cytometry. **(B,C)** Percentage and mean fluorescence intensity (MFI) of annexin V positive platelets upon activation with Spike or VSV-G pseudovirions. Results are from *n* = 4 independent experiments. Data are mean ± SEM. Statistical significance is indicated (paired Student’s *t*-test). AU, arbitrary units. **(D)** Representative flow cytometry plots. The boxed area shows the percentage of platelets positive for annexin V (AnV) binding following incubation with VSV-G or Spike. The histogram on the right shows the distribution of Annexin V positive platelets after the two treatments. For each flow cytometry plot, the first gating was based on CD61/FSC to define the platelet population. Thus, the Aneexin V plot excludes any microvesicles which may have been present within the sample. **(E)** Experimental scheme to study Ca^2+^ flux in platelets. Washed platelets were stained with Fluo-4 for 30 min, followed by incubation with VSV-G or Spike pseudovirions diluted 1:10 for 10 min. Platelet samples were then incubated with collagen (30 μg/ml) for 15 min and then analyzed by flow cytometry. **(F)** Mean fluorescence intensity (MFI) of Fluo-4 (AU, arbitrary units) in platelets stimulated with Spike or VSV-G pseudovirions. Results are from *n* = 4 independent experiments. Data are mean ± SEM. Statistical significance is indicated (paired Student’s *t*-test). **(G)** Representative flow cytometry plots. The boxed area shows the percentage of platelets positive for Fluo-4 fluorescence following incubation with VSV-G or Spike. The histogram on the right shows the distribution of Fluo-4 fluorescence after the two treatments. **(H)** Experimental scheme for the clot retraction assay following stimulation of platelets with Spike. PRP was supplemented with CaCl_2_ and 10 μl of whole blood, then incubated with 1:10 diluted VSV-G or Spike pseudoparticles, recombinant Spike or recombinant RBD (1 ng/mL) for 10 min at 37°C, followed by incubation with thrombin. Clot retraction was measured over 90 min, taking an image every 15 min. **(I,K)** Clot retraction of PRP pre-incubated with PBS, VSV-G or Spike pseudoparticles. Representative images immediately after the addition of thrombin and after 45 min are in panel **(I)**, the percentage of clot retraction over a 90 min observation period is in panel **(J)**. The graph in **(K)** shows the time to 50% clot retraction. All data are mean ± SEM from *n* = 5 independent experiments. Statistical significance is indicated in panel **(K)** (one-way ANOVA with Dunnett’s *post-hoc* correction for multiple comparisons). **(L,M)** Clot retraction of PRP pre-incubated with PBS, recombinant Spike (1 ng/mL) or recombinant receptor binding domain (RBD, 1 ng/mL). The percentage of clot retraction over the 90 min observation period is in panel **(L)**. The graph in panel **(M)** shows the time to 50% clot retraction in the three experimental conditions. All data are mean ± SEM from *n* = 3 independent experiments. Statistical significance is indicated in panel **(K)** (one-way ANOVA with Dunnett’s *post-hoc* correction for multiple comparisons). **(N)** Experimental scheme to study thrombin generation upon platelet treatment with Spike pseudovirions. PRP was incubated with 1:10 diluted VSV-G or Spike pseudovirions for 10 min at 37°C, followed by stimulation with collagen (30 μg/mL) for 15 min (350 rpm, 37°C) and the addition of a fluorogenic thrombin substrate. Thrombin activity was assessed by measuring the conversion of thrombin substrate into its fluorogenic state using a CLARIOstar fluorescent plate reader with 350/450 nm excitation and emission filter. Analysis was performed using MARS analysis software. **(O)** Concentration of thrombin formed during a 30 min-time period. Results are from *n* = 5 independent experiments. Data are mean ± SEM.

As Spike activates TMEM16F (36) and this scramblase stimulates the procoagulant activity of platelets (40–42), we wanted to study platelet PS externalization upon treatment with Spike (Figure 2A). We observed that the dual stimulation of platelets with collagen and thrombin in the presence of Spike virions increased both the percentage of annexin V-positive platelets (7.0 vs. 10 ± 1% in VSV-G vs. Spike; *n* = 4, *P* < 0.05) and the amount of platelet-bound annexin V [1,524 ± 98 vs. 1,755 ± 82 arbitrary units (AU) in VSV-G vs. Spike; *n* = 4, *P* < 0.01; Figures 2B, C, respectively; representative flow cytometry profiles are in Figure 2D].

Given the relevance of Ca^2+^ signaling in platelet activation and procoagulant activity, next we investigated the effect of Spike on Ca^2+^ flux (Figure 2E). We observed that, in platelets activated with collagen, Spike increased the levels of the Fluo-4 indicator, which is sensitive to cytosolic Ca^2+^ (16,756 ± 2,115 AU vs. 19,281 ± 1,952 AU in VSV-G vs. Spike; *n* = 4, *P* < 0.05; Figure 2F; representative flow cytometry plots are in Figure 2G). This increase was only observed in the presence of extracellular Ca^2+^ (Supplementary Figure 2). This indicates that Spike-mediated platelet activation is not related to Ca^2+^ release from the platelet intracellular stores or, should release occur, this is not amplified and sustained by store-operated Ca^2+^ entry.

Considering these results, we wanted to assess whether Spike also affected clot formation and retraction, which are the last steps in the coagulation cascade (Figure 2H). We observed that Spike markedly enhanced thrombin-induced clot retraction (representative images in Figure 2I; complete time point set in Supplementary Figure 3). The kinetics of clot formation in the presence of Spike was faster compared to both VSV-G-treated or control platelets (Figure 2J; difference in Area Under the Curve, AUC: *P* < 0.001 for both controls), with a time to 50% clot retraction of 16 ± 5 min compared to 42 ± 5 min after treatment with VSV-G; *n = 4, P* < 0.05 (Figure 2K). The same result was also obtained by measuring the clot retraction after incubating platelets with recombinant proteins corresponding to full-length Spike or to the Spike receptor binding domain (RBD) (Figures 2L, M for kinetic analysis and time to 50% clot retraction, respectively; *P* < 0.05 in both cases).

We also observed that the kinetics of thrombin generation following stimulation with collagen (scheme in Figure 2N) was markedly increased by incubation with the Spike virions (1.5 ± 0.4 ng vs. 19.4 ± 3.1 ng thrombin concentration in VSV-G vs. Spike at 30 min; *n* = 5, AUC difference: *P* < 0.01; Figure 2O). There was no significant difference between untreated and VSV-G-treated platelets at any time point (not shown).

Collectively, these results indicate that Spike increases platelet Ca^2+^ flux, PS exposure and thrombin-induced clot retraction, all of which are markers of the procoagulant function in platelets.

### 3.3 Drugs inhibiting Spike-induced syncytia formation also block the effect of Spike on platelets

**FIGURE 3:**
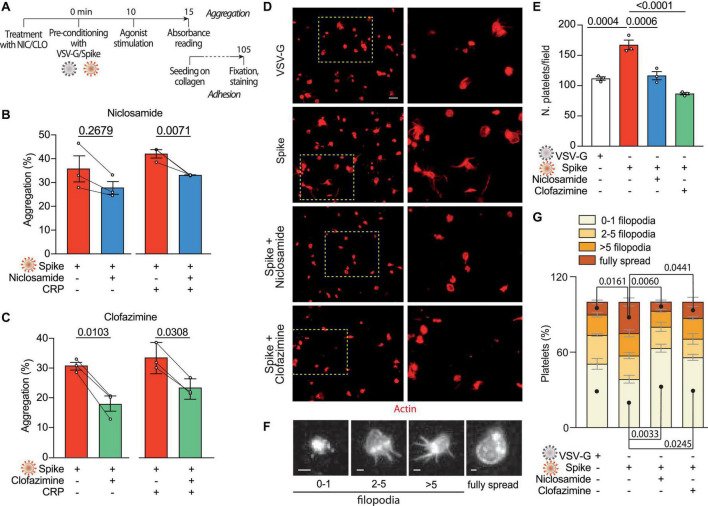
Niclosamide and Clofazimine inhibits Spike-induced activation of platelets. **(A)** Experimental scheme to assess the effect of drugs on platelet activation. Washed platelets were incubated with Niclosamide, Clofazimine **(C)** or vehicle for 10 min, then incubated with 1:10 diluted Spike or VSV-G pseudoparticles for further 10 min. Aggregation and adhesion were evaluated as described in Figure 1. **(B,C)** Percentage of platelet aggregation upon treatment with Spike in the presence of either Niclosamide **(B)** or Clofazimine **(C)**, and respective DMSO controls, with or without CRP. Results are from *n* = 3 independent experiments. Data are mean ± SEM. Statistical significance is indicated (paired Student’s *t*-test). **(D)** Representative images of platelets adhering on collagen (magnification in the right panels). Platelets are stained in red for F-actin. Scale bar, 5 μm. **(E)** Number of adherent platelets per field. Results are from *n* = 3 independent experiments performed in duplicate. Each dot represents the mean of six quantified images. Data are mean ± SEM. Statistical significance is indicated (unpaired Student’s *t*-test). **(F)** Representative images of platelet morphological changes. Adherent platelets were classified into four morphological categories representing different stages of platelet adhesion and activation: platelets with 0–1, 3–5, more than five protrusions or fully spread platelets, as indicated by the representative images under the graph. Scale: 1 μm. **(G)** Platelet morphological changes upon Spike and drug treatment. Results are from *n* = 3 independent experiments performed in duplicate. Data are mean ± SEM. Statistical significance is shown for the indicated morphological categories (one-way ANOVA with Dunnett’s *post-hoc* correction for multiple comparisons).

Our previous screening work has identified Niclosamide and Clofazimine as the two most potent drugs that inhibit Spike-induced cell fusion by targeting TMEM16F (36). We confirmed that this scramblase is well expressed in platelets (immunoblotting from six normal donors in Supplementary Figure 4A) and thus hypothesized that Niclosamide and Clofazimine could inhibit Spike-induced platelet activation (scheme in Figure 3A).

Pre-treatment of platelets with either drug, followed by incubation with VSV-G or Spike pseudotyped particles, reduced platelet aggregation upon stimulation with CRP (shown in Figure 3B for Niclosamide—from 42 ± 2 to 33%; *P* < 0.05, and in Figure 3C for Clofazimine; from 34 ± 3 to 23 ± 2%; *P* < 0.01). Clofazimine also inhibited aggregation in the absence of CRP treatment (from 31 ± 1 to 18 ± 3%; *P* < 0.01). There was no effect when the drugs were used in the presence of the VSV-G pseudotypes (Supplementary Figures 4B, C).

We next sought to understand whether Niclosamide and Clofazimine affected Spike-induced platelet adhesion on collagen. Pre-treatment with either drug significantly reduced the total number of adherent platelets (representative images and quantification in Figures 3D, E; 169 ± 9 platelets/field in vehicle-treated samples vs. 118 ± 7 and 88 ± 2 platelets/field in Niclosamide and Clofazimine-treated platelets, respectively; *n* = 3, *P* < 0.01 in both cases). Pre-treatment with neither Niclosamide nor Clofazimine affected platelet adhesion in VSV-G controls (Supplementary Figure 4D).

Finally, we assessed the spreading stage of adherent platelets as an indicator of drug efficacy. In VSV-G-treated samples, most platelets showed a rounded shape with no or single filopodia (filopodia patterns are in Figure 3F). Platelet activation by Spike increased the percentage of platelets with multiple filopodia and of platelets showing a fully spread phenotype (Figure 3G). Treatment with either Niclosamide and Clofazimine reversed this Spike-induced phenotype (*n* = 3 per condition; *P* < 0.05 for both platelet phenotype distribution between VSV-G and Spike and for the effect of either drug vs. Spike).

### 3.4 Niclosamide and Clofazimine block Spike-induced platelet procoagulant activity

**FIGURE 4:**
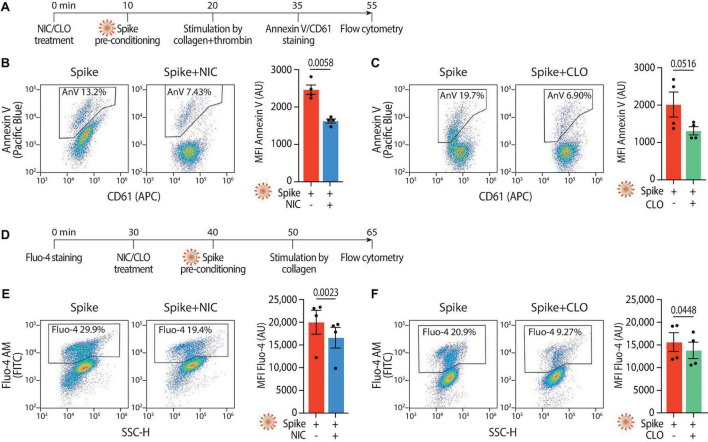
Niclosamide and Clofazimine reduce annexin V reactivity and intracellular calcium in platelets. **(A)** Experimental scheme to assess annexin V reactivity upon Spike stimulation and drug treatment. Platelets were pre-incubated with Niclosamide (NIC, 1 μM) or Clofazimine (CLO, 5 μM) for 10 min, followed by incubation with collagen (30 μg/ml) and thrombin (0.5 units) for 15 min. Platelets were then stained with Annexin V-Pacific Blue and CD61-APC and analyzed by flow cytometry. **(B)** Effect of Niclosamide on annexin V reactivity. Representative flow cytometry plots are on the left, quantification on the right. The boxed areas show the percentage of washed platelets positive for annexin V upon treatment with the drug or vehicle (DMSO at the same concentration and for the drug) and incubation with VSV-G or Spike pseudoparticles, followed by stimulation with collagen and thrombin. The graph shows the Mean fluorescence intensity (MFI) of annexin V positive platelets (AU, arbitrary units). Results are from *n* = 4 independent experiments. Data are mean ± SEM. Statistical significance is indicated (paired Student’s *t*-test). **(C)** Same as panel **(B)** upon treatment with Clofazimine. **(D)** Experimental scheme to assess Ca^2+^ influx upon Spike stimulation and drug treatment. Platelets were stained with Fluo-4 for 30 min and then pre-incubated with Niclosamide (NIC, 1 μM) or Clofazimine (CLO, 5 μM) for 10 min, followed by incubation with collagen (30 μg/ml) and thrombin (0.5 units) for 15 min. Platelets were then assessed for fluorescence by flow cytometry. **(E)** Representative flow cytometry plots are on the left, quantification on the right. The boxed areas show the percentage of washed platelets positive for Fluo-4 upon treatment with the drug or vehicle (DMSO at the same concentration and for the drug) and incubation with Spike pseudoparticles, followed by stimulation with collagen and thrombin. The graph shows the Mean fluorescence intensity (MFI) of Fluo-4 (AU, arbitrary units). Results are from *n* = 4 independent experiments and are expressed as mean ± SEM. Statistical significance is indicated (paired Student’s *t*-test). **(F)** Same as in panel **(E)** upon treatment with Clofazimine.

Given the reduction in aggregation and adhesion, we next investigated whether Niclosamide and Clofazimine also inhibited the procoagulant response to Spike (scheme in Figure 4A). Pre-treatment of platelets with either drug reduced PS exposure (2,467 ± 125 Annexin V AU vs. 1,627 ± 55 AU in Spike vs. Niclosamide for mean fluorescent intensity; 2,064 ± 343 AU vs. 1,341 ± 113 AU in Spike vs. Clofazimine; *n* = 4, *P* < 0.05 in both cases; representative flow cytometry plots and quantification in Figures 4B, C; the difference in the percentages of fluorescent cells was also statistically different for both drugs–not shown). The levels of Spike-induced intracellular Ca^2+^ were reduced by both drugs (scheme in Figure 4D; Fluo-4 fluorescence: 20,014 ± 2,627 AU vs. 16,607 ± 2,278 AU in Spike vs. Niclosamide for mean fluorescent intensity; 15,605 ± 2,083 AU vs. 13,804 ± 1,816 AU in Spike vs. Clofazimine; *n* = 4, *P* < 0.05 in both cases; Figures 4E, F). In a consistent manner and for both drugs, the percentages of fluorescent cells also showed statistically significant differences (not shown).

**FIGURE 5:**
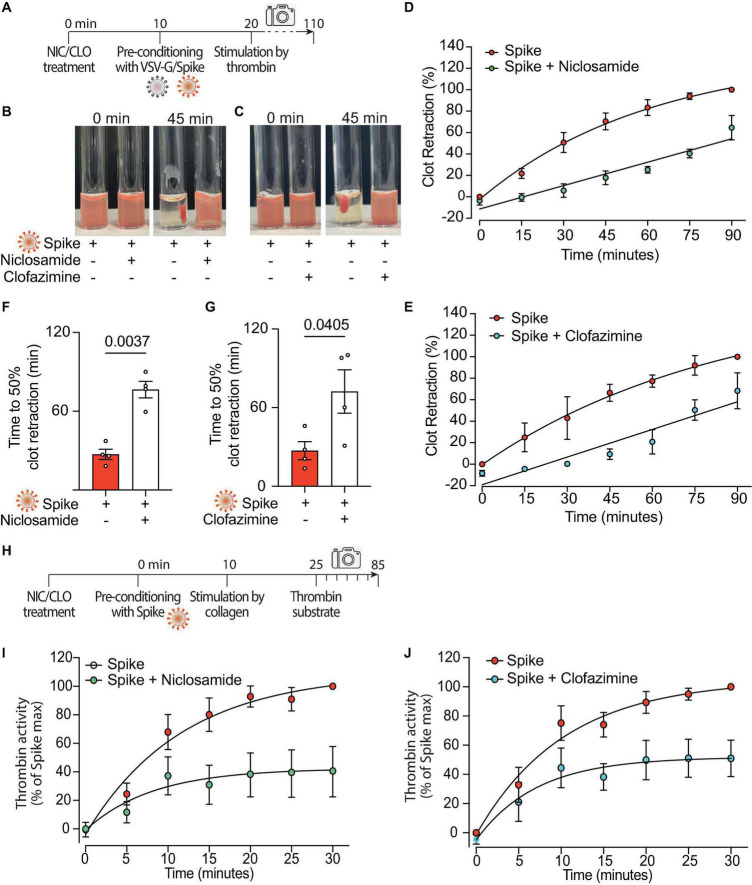
Niclosamide and Clofazimine block procoagulant activation of platelets. **(A)** Experimental scheme to study the effect of drugs on the clot retraction assay following stimulation of platelets with Spike. PRP, supplemented with CaCl_2_ and 10 μl of whole blood, were incubated with Niclosamide or Clofazimine for 10 min, then treated with 1:10 diluted VSV-G or Spike pseudoparticles for additional 10 min. Clot retraction was measured over 90 min from the addition of thrombin, taking an image every 15 min. **(B,C)** Representative images of clot retraction when platelets were treated with Spike and either Niclosamide **(B)** or Clofazimine **(C)**, immediately after the addition of thrombin and after 45 min. **(D,E)** Percentage of clot retraction over 90 min, when platelets were treated with Spike and either Niclosamide **(J)** or Clofazimine **(K)**. Results are from *n* = 4 independent experiments. Data are expressed as mean ± SEM. **(F,G)** Time to 50% clot retraction when platelets were treated with Spike and either Niclosamide **(L)** or Clofazimine **(M)**. Results are from *n* = 4 independent experiments. Data are mean ± SEM. Statistical significance is shown (paired Student’s *t*-test). **(H)** Experimental scheme to study the effect of drugs on thrombin generation upon platelet treatment with Spike pseudovirions. PRP was incubated with 1:10 diluted VSV-G or Spike for 10 min at 37°C, followed by incubation with collagen (30 μg/mL) for 15 min and the addition of a fluorogenic thrombin substrate. Thrombin activity was assessed by the measurement of the conversion of the thrombin substrate into its fluorogenic state. **(I,J)** Concentration of thrombin formed during a 30 min-time period in PRP treated with Spike and pre-conditioned or not with Niclosamide or Clofazimine. Data are expressed as mean ± SEM.

Both Niclosamide and Clofazimine reduced the rate of thrombin-stimulated clot retraction (experimental scheme in Figure 5A, representative images in Figures 5B, C, respectively; complete time courses in Supplementary Figures 5A, D). In the presence of either drug, the kinetics of Spike-induced clot formation was reduced (Figures 5D, E, respectively; AUC difference: *P* < 0.001 and *P* < 0.05 for the two drugs, respectively). The time to 50% clot retraction increased from 27 ± 4 min to 76 ± 6 min in the presence of Niclosamide and from 27 ± 7 min vs. 72 ± 17 min in the presence of Clofazimine (*n* = 3, *P* < 0.05 in both cases; Figures 5F, G). Of interest, Niclosamide had no significant effect on the rate of thrombin-stimulated clot retraction in the absence of Spike, namely upon addition of PBS or treatment with VSV-G pseudovirions (Supplementary Figures 5B, C, respectively). This is consistent with the conclusion that Niclosamide specifically blocks Spike-induced platelet activation through TMEM16F, in agreement with its role as a specific TMEM16F inhibitor. In the case of Clofazimine, partial inhibition of thrombin-stimulated clot retraction was also observed in the absence of Spike (Supplementary Figures 5E, F), albeit at a lower extent than upon stimulation with Spike. This could indicate that Clofazimine also acts through additional platelet activation pathways.

Finally, we assessed the effect of the two drugs on the kinetics of thrombin generation following stimulation with collagen in the presence of virions with Spike (scheme in Figure 5H). Both drugs markedly decreased thrombin formation (62% decrease vs. Spike at 30 min for Niclosamide, 55% decrease for Clofazimine; *n* = 5, *P* < 0.01 at all time points from 10 to 30 min; Figures 5I, J; AUC difference: *P* < 0.01 in both cases).

## 4 Discussion

There is broad evidence of platelet dysregulation during SARS-CoV-2 infection, by which platelets are hyperreactive in several of their phenotypes. Manne et al. reported that platelets from COVID-19 patients aggregate faster, showing increased spreading over fibrinogen/collagen–coated surfaces and increased P-selectin expression, despite integrin activation was reduced (31). Zaid et al. observed that platelets from COVID-19-positive patients showed increased aggregation, adhesion, and microvesiculation to subthreshold concentration of thrombin (32). Nicolai et al. reported that cases of intermediate severity showed an exhausted platelet phenotype, while patients severely affected with COVID-19 were characterized by excessive platelet activation in comparison with healthy controls and non-COVID-19 pneumonia (46). Finally, Zhang et al. observed platelet hyperreactivity based on increased integrin αIIbβ3 activation and P-selectin expression (47).

Here we show that exposure of platelets to SARS-CoV-2 Spike promotes platelet activation and adhesion, and enhances Ca^2+^ release and PS exposure to drive increased thrombin generation. Externalized, negatively charged PS can act as a platform for assembly of the tenase and prothrombinase complex, which massively amplify thrombin generation (42). In addition, increased PS exposure could contribute to inflammation and immune reactivity. These phenotypes in platelets, and in particular the procoagulant activity of Spike, were blocked by Niclosamide, a TMEM16A and TMEM16F inhibitor (48). The relevance of TMEM16F for the procoagulant phenotype of platelets is underscored by the phenotypes of Scott patients [reviewed in Nurden et al. (49)] and PF4-Cre-conditioned TMEM16F-null mice (41), both of which have a normal platelet count and do not show obvious defects in platelet activation but indeed display a reduced procoagulant phenotype in association with reduced thrombin generation.

The effects of Spike were present when we used this protein as a sole agonist, but was markedly enhanced in the presence of known platelet activators. This observation suggests that Spike, in the lungs of severely infected individuals, could contribute to the pro-thrombotic state, by cooperating with hyperinflammation (15–19), endothelial dysfunction (6, 14) and NETosis (23, 24) in determining thrombosis. This could explain the prevalence of thrombosis in the lungs of COVID-19 patients with severe disease, which is significantly higher than in other forms of ARDS.

In our experiments, SARS-CoV-2 Spike stimulated platelets both when present on the virion envelope or upon expression on the cell plasma membrane. When and in what compartment would platelet become in direct contact with the SARS-CoV-2 Spike protein in patients? There are at least three possibilities. First, through direct viral infection of endothelial cells. There is evidence of endothelial cell infection by SARS-CoV-2 by viral RNA analysis (7, 14), while we and others have detected Spike by immunostaining on endothelial cells in SARS-CoV-2 lungs of COVID-19 patients at post-mortem analysis (6, 50, 51). Second, SARS-CoV-2 can be found in the blood (52) and viral production is particularly robust in the lung and lower tract respiratory epithelium of SARS-CoV-2 infected patients, leading to the continuous production of infectious particles (53). Our own work has shown that SARS-CoV-2 RNA is detectable in plasma or serum of COVID-19 ICU patients when neutralizing antibody response is low. RNAemia was associated with higher 28-day ICU mortality (54). Third, and probably most relevant, the hyperinflammatory environment in the infected lungs can promote the disruption of the endothelial barrier, which would allow platelets to enter the infected lung tissue and get in contact with infected pneumocytes. These infected cells (36) persist for prolonged periods during infection while expressing viral antigens, including Spike. Cell surface expression of Spike leads to fusion of the infected cells with neighboring cells expressing the ACE2 receptor, as SARS-CoV-2 Spike, in contrast to the homologous protein from SARS-CoV, contains a furin cleavage site that allows protein activation while the protein is produced during the ER-Golgi transit or when at the cell surface (55–57). The syncytia formed because of these characteristics can be found in over 90% of patients with severe infection and express detectable amounts of Spike on their surface (36). These infected cells and syncytia represent a platform for platelet activation and the consequent induction of thrombosis.

In our experiments, Spike was also active when administered as a recombinant protein, which raises the question as to what the mechanism is for Spike-mediated platelet activation. Based on our previous observations on the activation of TMEM16F by Spike (36), we can envisage at least two mechanistic possibilities. Activation could occur directly upon binding of Spike to its receptor, leading to the direct activation of TMEM16F on the platelet membrane. Alternatively, activation of TMEM16F could be triggered by the increase on Ca^2+^ we observe after stimulation with Spike. In other cell types, Spike-mediated TMEM16 activation increases the amplitude of spontaneous Ca^2+^ signals (36), which is in line with previous reports showing that both TMEM16A and TMEM16F augment intracellular Ca^2+^ by increasing the filling of ER stores and augmenting IP3R-induced Ca^2+^ release (58). Two of the experiments that we report here, however, favor the former possibility, namely that Spike-induced platelet activation is consequent to a molecular event occurring at the membrane level. First, activation of platelets did not occur when the medium was depleted of extracellular Ca^2+^, likely indicating that intracellular Ca^2+^ stores are not required for activation. Second, and most important, platelet activation also occurred upon treatment with the isolated spike RBD domain. These observations are in favor of a direct TMEM16F activation event occurring upon binding of Spike at the plasma membrane level.

As far as the platelet Spike-binding receptor is concerned, this remains still controversial. A couple of reports failed to detect ACE2 in platelets from both COVID-19 patients and healthy individuals (31, 32). Other investigators, however, have reported expression of ACE2 on platelets from both healthy individuals and mice by RT-PCR (47), and also expression of the ACE2 protein by immunodetection (59). In our own experiments, we could readily detect expression of ACE2 in the healthy donors we analyzed, from whom platelets were collected for our experiments. Irrespective of the actual expression of ACE2, however, all the studies so far conducted, including those reporting against ACE2 expression, are concordant in showing platelet activation in SARS-CoV-2 infection, and several of these studies have also confirmed that the viral genome can be found within platelets (31, 32, 59). Should ACE2 not be the relevant receptor, there may be ACE2-independent mechanisms whereby SARS-CoV-2 directly interacts with, and possibly enters, platelets [reviewed in ref: (60)].

Both Niclosamide and Clofazimine were remarkably effective at inhibiting Spike-induced platelet activation. In the case of Niclosamide, this in line with the inhibition of TMEM16F and the known role that this protein plays in amplifying the rate of platelet procoagulant activity (42). Niclosamide acted at concentrations in the low hundred nanomolar range, which are even lower than those needed to inhibit syncytia (36). Niclosamide is a synthetic salicylanilide developed in the 1950s as a molluscicide against snails (37) and subsequently approved for use in humans, where it has been employed for over 50 years to treat tapeworm infections (61). Solubility of the currently available oral formulation is relatively low, but there is anyhow evidence of significant systemic absorption, with plasma levels that can reach 1–20 μM (62). Besides inhibiting TMEM16 proteins, multiple evidence indicates that this drug can exert pleiotropic effects in mammalian cells, which include modulation of the Wnt/β-catenin and Notch signaling pathways, repression of mTORC1 and inhibition of STAT3 and NF-κB transcriptional regulators [reviewed in Chen et al. (63)]. Some of these various functions could explain the effects of Niclosamide on platelet aggregation, adhesion and spreading, which are not directly relatable to TMEM16F activation. In this respect, however, it should be noted that the magnitude of these effects, albeit their statistical significance, is markedly lower than that of the inhibition of platelet pro-coagulant activity.

Finally, an interesting open question is whether the mechanism for Spike-induced platelet activation could be causally linked to the thrombocytopenia in the course of COVID-19 (5, 25–27). Decreased platelet levels due to clearance in the spleen are reported in other conditions of PS overexposure (64), including mice with reduced levels of Bcl-xL (64). Recent evidence links this feature specifically to TMEM16F and extracellular Ca^2+^ influx, as mice with sphingomyelin synthase one deficiency show marked thrombocytopenia due to increased PS exposure consequent to excessive TMEM16F activation (65). Based on these observations, we speculate that thrombocytopenia during severe COVID-19 may be consequent to abnormal platelet activation through Spike-mediated TMEM16F stimulation.

## Data availability statement

The original contributions presented in this study are included in the article/Supplementary material, further inquiries can be directed to the corresponding authors.

## Ethics statement

The studies involving human participants were reviewed and approved by the Joint Ethical Committee of the Regione Friuli Venezia Giulia, Italy (re. 0019072/P/GEN/ARCS). The patients/participants provided their written informed consent to participate in this study.

## Author contributions

AC, ES, HA, and IS performed the molecular and cellular biology experiments and generated pseudovirions. HEA and MC performed experiments with human platelets. SV, RS, and LM participated in experiments on Spike-expressing cells. SZ contributed to conceiving the project. TW and MG conceived the project, coordinated the work, and wrote the manuscript. All authors read and edited the manuscript.
