# Supplementary material for: SARS-CoV-2 Spike protein activates TMEM16F-mediated platelet procoagulant activity
Source: Front Cardiovasc Med. 2023 Jan 4;9:1013262. doi: 10.3389/fcvm.2022.1013262 (PMC9845929; doi:10.3389/fcvm.2022.1013262)
Supplement: Supplementary file 1 [file Data_Sheet_1.PDF]

SARS-CoV-2 Spike protein activates TMEM16F-mediated platelet procoagulant activity
A. Cappelletto et al.

SUPPLEMENTARY FIGURES

SUPPLEMENTARY FIGURE LEGENDS

Supplementary Figure 1. Characterisation and effect of lentiviral vectors pseudotyped with SARS-CoV-2 Spike or VSV-G.

A. Expression of ACE2 in platelets from normal donors. RNA was extracted from washed platelet preparations and submitted to qRT-PCR using primers and probes from Dharmacon. Data (mean±SD of three independent replicates) are expressed after normalisation for the cellular 18S RNA and as fold over ACE2 mRNA levels in respiratory Calu-3 cells.

B. Visualisation of ACE2 (magenta; with 2 different antibodies, as indicated) and TMEM16F (blue) proteins by immunofluorescence in platelets from two donors. Platelets are also stained with an antibody against tubulin (green). Scale bar: 5 µm.

C. Viral genome titration of pseudoparticles. Results are from the quantifications of n=3 pseudoparticle preparations.

D. Immunoblot showing the expression of Spike in pseudoparticles. Blots were incubated (4°C, overnight) with a primary antibody recognizing Spike (Genetex, 1:1,000) followed by incubation with an anti-mouse HRP-conjugated antibody (1:10,000) and subsequent development with ECL (Amersham).

E, F. HEK-293T cells overexpressing the human ACE2 were treated with VSV-G or Spike pseudoparticles and fixed after 48 hr. Percentage of transduced cells (E) and representative images (F). In green, GFP; in blue, Hoechst. Scale bar, 20 µm. SARS-CoV-2 Spike pseudotyped vectors are known to have a lower efficiency compared to VSV-G-pseudotyped vectors, despite the use of the Δ19 C-terminal deletion, which increases transport of Spike to the plasma membrane (Cattin-Ortola J et al. Nat Commun 2021;12:5333).

G, H. Platelets were incubated either with VSV-G or PBS for 10 min at 200 rpm, at 37°C and then treated with PBS (G; unstimulated) or stimulated with CRP (H; final concentration 0.3 µg/mL). Aggregation was measured as described in Figure 1. Results are from n=3 independent experiments. Data are mean±SEM. Statistical significance is shown (paired Student's t-test).

I. Platelet adhesion was determined as described in Figure 1. Results are from n=3 independent experiments. Data are mean±SEM. Statistical significance is shown (paired Student's t-test).

Supplementary Figure 2. Calcium influx in control conditions

Mean fluorescence intensity (MFI) and percentage of positive cells (**A** and **B** respectively) of annexin V positive platelets upon addition of buffer (Control) or VSV-G pseudovirions. Results are from n=4 independent experiments. Data are mean±SEM. Statistical significance is indicated (paired Student's t-test). AU: arbitrary units. **C.** Mean fluorescence intensity (MFI) of Fluo-4 (AU, arbitrary units) in platelets stimulated with Spike or VSV-G

pseudovirions the absence of extracellular calcium. Washed platelets were stained with Fluo-4 for 30 min, followed by incubation with 1:10 diluted VSV-G or Spike pseudoparticles for additional 10 min. Platelet samples were activated with collagen (30 µg/ml) for 15 min and then analysed by flow cytometry. Results are from n=2independent experiments. Data are means.

Supplementary Figure 3. Clot retraction assay

Images of a representative clot retraction assay using PRP incubated with Niclosamide or Clofazimine for 10 min, followed by treatment with 1:10 diluted VSV-G or Spike pseudovirions for additional 10 min. The images were taken every 15 min. P, PBS; V, VSV-G; S, Spike.

Supplementary Figure 4. Expression of TMEM16F in platelets and effect of drugs and VSV-G pseudovirions

A. Immunoblot showing the expression of TMEM16F by washed platelets (WP) from different normal individuals. Blots were then incubated (4°C, overnight) with primary antibodies recognizing TMEM16F (1:1,000) and tubulin (1:10,000), followed by incubation for 1 hr with either anti-rabbit HRP-conjugated antibody (1:5,000) or anti-mouse HRP-conjugated antibody (1:10,000). ECL (Amersham) was used for blot development.

B. Platelets were incubated either with Niclosamide or PBS for 10 min, then treated with either VSV-G pseudoparticles or PBS for additional 10 min. Aggregation was measured as described in Figure 3. Results are from n=3 independent experiments. Data are mean±SEM. Statistical significance is shown (paired Student's t-test).

C. Platelets were incubated either with Clofazimine or PBS for 10 min, then treated with either VSV-G pseudoparticles or PBS for additional 10 min. Aggregation was measured as described in Figure 3. Results are from n=3 independent experiments. Data are mean±SEM. Statistical significance is shown (paired Student's t-test).

D. Platelet adhesion was evaluated as described in Figure 3. Results are from n=3 independent experiments performed in duplicate; 6 images per well were analysed. Data are mean±SEM. Statistical significance is shown (one-way ANOVA with Dunnett's multiple comparison test).

Supplementary Figure 5. Effect of PBS and VSV-G Controls on clot retraction

A. Images of a representative clot retraction assays using PRP incubated with Niclosamide or Clofazimine for 10 min, then treated with 1:10 diluted VSV-G or Spike for 10 min. Images were taken every 15 min. P, platelets; V, VSV-G; S, Spike; N, Niclosamide; C, Clofazimine.

B-C. Graphs showing the percentage of clot retraction over a 90 min observation, upon PRP treatment with either PBS or VSV-G (B and C respectively) in the presence or absence of Niclosamide. Results are from n=4 independent experiments. Data are mean ± SEM.

D-F. Same as panels A-C using Clofazimine.

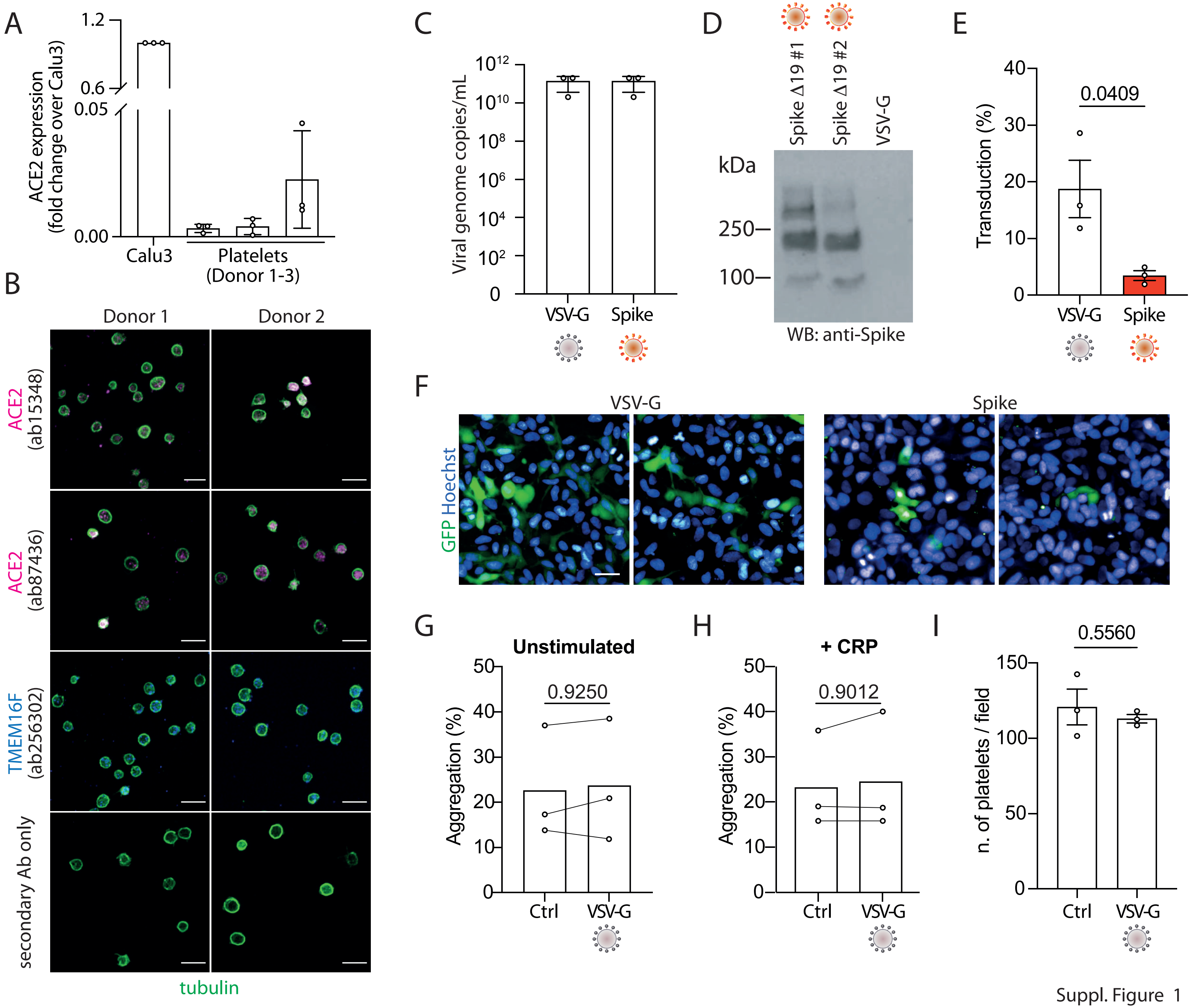

Suppl. Figure 1

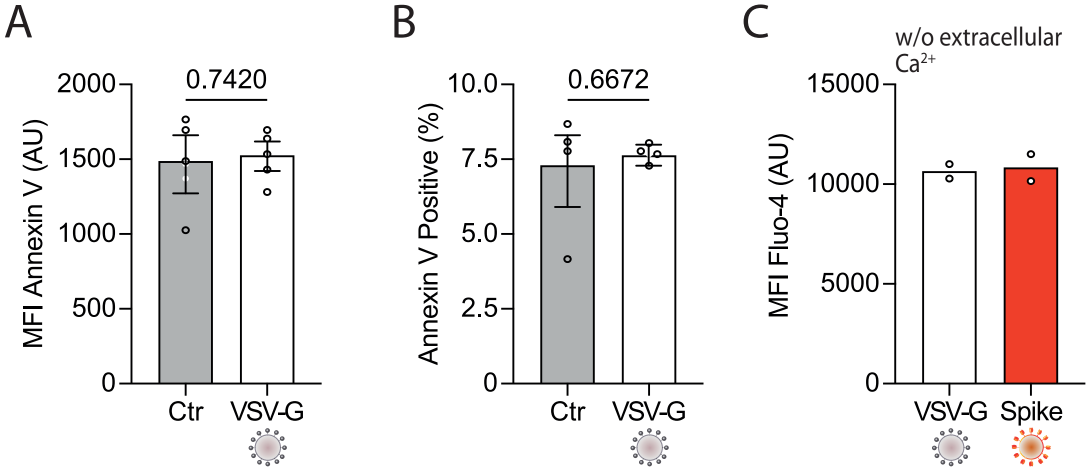

Suppl. Figure 2

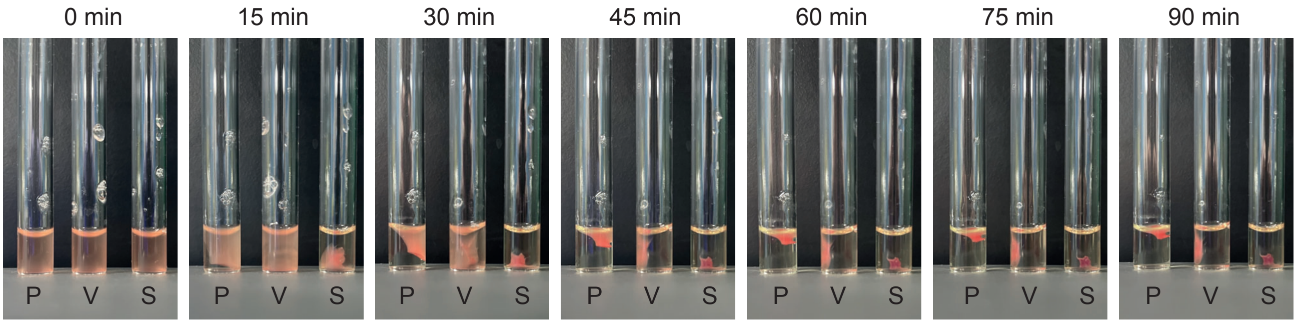

P = Ctrl (PBS)
V = VSV-G pseudoparticles
S = Spike pseudoparticles

Suppl. Figure 3

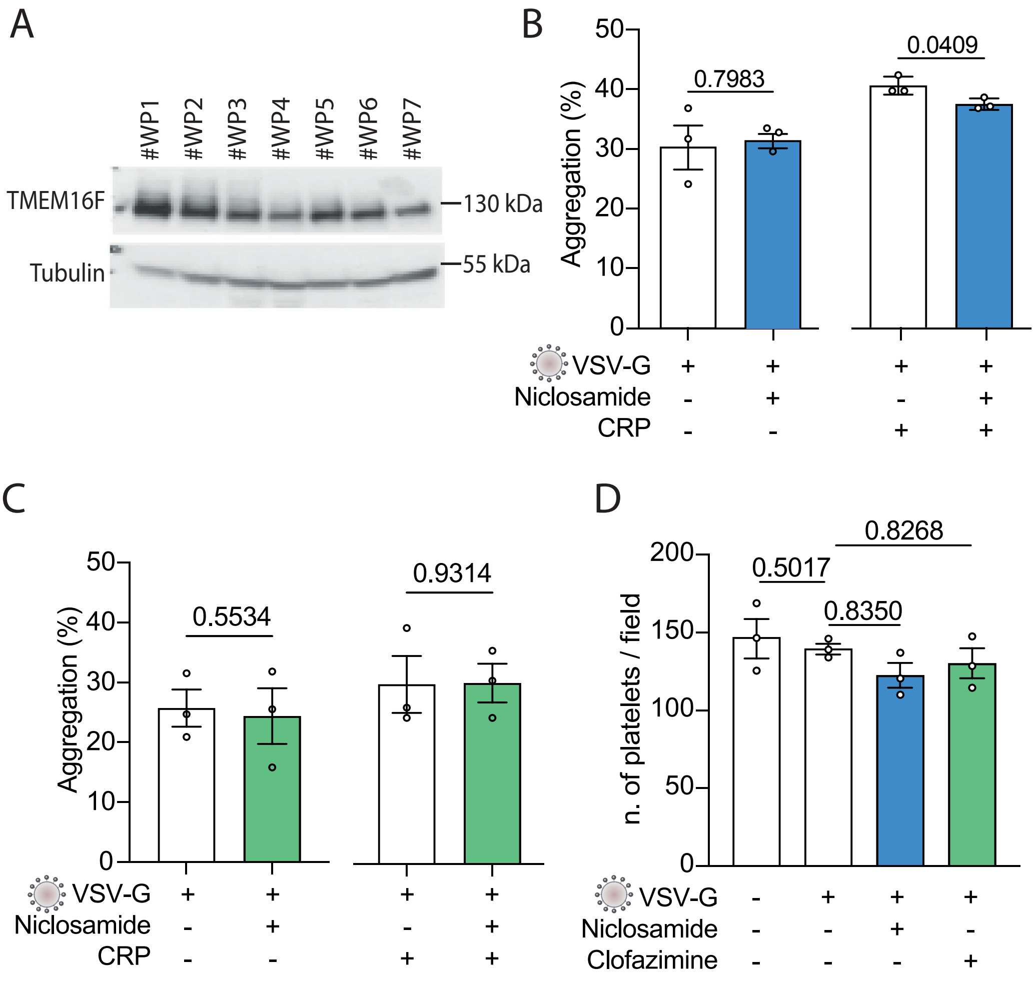

Suppl. Figure 4

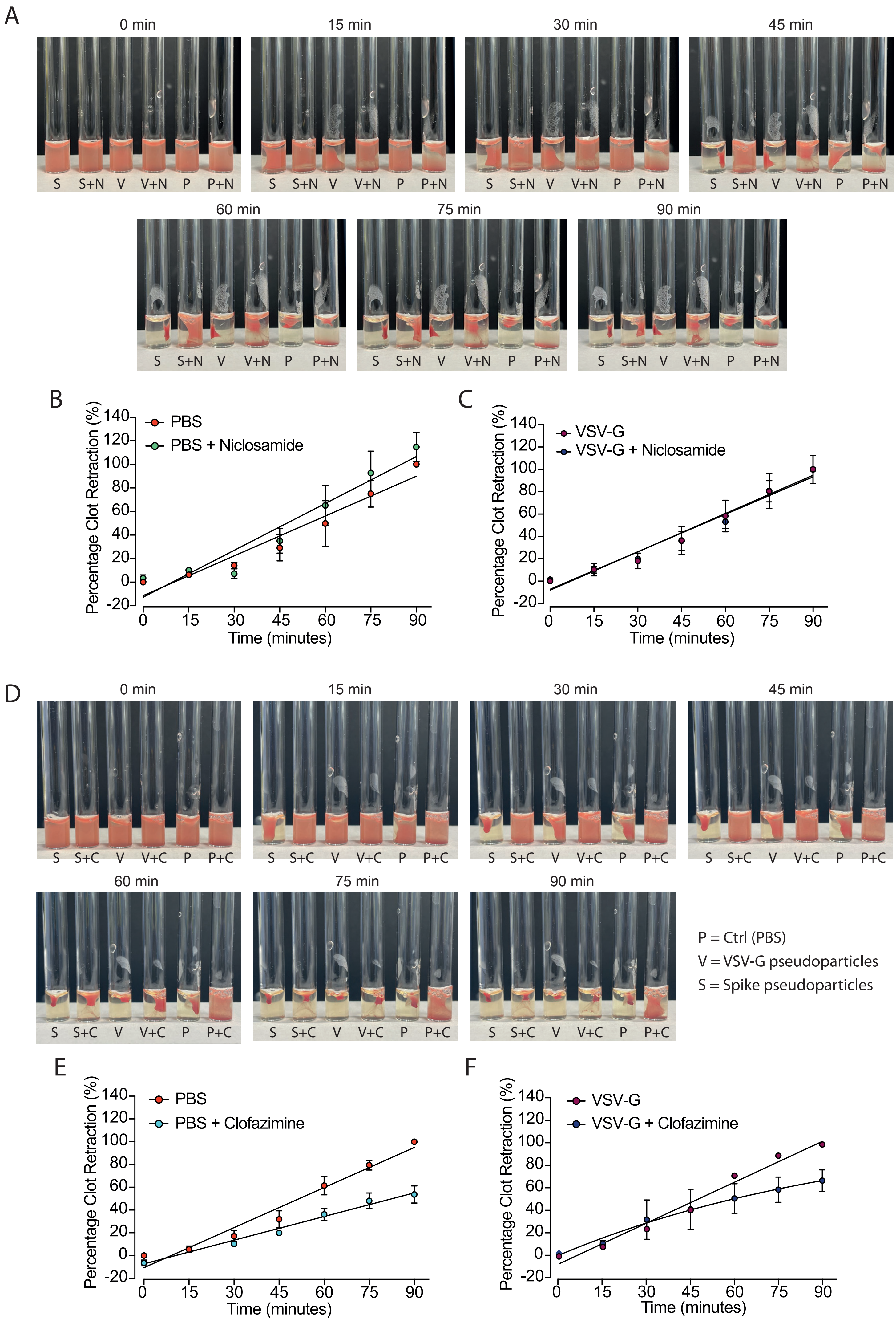

Suppl. Figure 5
